# Priming with a Recombinant Pantothenate Auxotroph of *Mycobacterium bovis* BCG and Boosting with MVA Elicits HIV-1 Gag Specific CD8^+^ T Cells

**DOI:** 10.1371/journal.pone.0032769

**Published:** 2012-03-29

**Authors:** Rosamund Chapman, Enid Shephard, Helen Stutz, Nicola Douglass, Vasan Sambandamurthy, Irene Garcia, Bernhard Ryffel, William Jacobs, Anna-Lise Williamson

**Affiliations:** 1 Institute of Infectious Disease and Molecular Medicine, University of Cape Town, Cape Town, South Africa; 2 Division of Medical Virology, Department of Clinical Laboratory Science, University of Cape Town, Cape Town, South Africa; 3 Department of Medicine, Faculty of Health Sciences, University of Cape Town, Cape Town, South Africa; 4 Medical Research Council, Cape Town, South Africa; 5 AstraZeneca Research and Development, Infection iMED, Bangalore, India; 6 Department of Pathology and Immunology, Centre Médical Universitaire, Hôpitaux Universitaires de Genève, University of Geneva, Geneva, Switzerland; 7 University of Orleans and Centre National de la Recherche Scientifique, Unité Mixte de Recherche, Molecular Immunology and Embryology, Orleans, France; 8 Howard Hughes Medical Institute, Albert Einstein College of Medicine, Bronx, New York, United States of America; 9 National Health Laboratory Service, Cape Town, South Africa; Statens Serum Institute, Denmark

## Abstract

A safe and effective HIV vaccine is required to significantly reduce the number of people becoming infected with HIV each year. In this study wild type *Mycobacterium bovis* BCG Pasteur and an attenuated pantothenate auxotroph strain (BCGΔ*panCD*) that is safe in SCID mice, have been compared as vaccine vectors for HIV-1 subtype C Gag. Genetically stable vaccines BCG[pHS400] (BCG-Gag) and BCGΔ*panCD*[pHS400] (BCGpan-Gag) were generated using the Pasteur strain of BCG, and a panothenate auxotroph of Pasteur respectively. Stability was achieved by the use of a codon optimised *gag* gene and deletion of the *hsp60-lysA* promoter-gene cassette from the episomal vector pCB119. In this vector expression of *gag* is driven by the *mtrA* promoter and the Gag protein is fused to the *Mycobacterium tuberculosis* 19 kDa signal sequence. Both BCG-Gag and BCGpan-Gag primed the immune system of BALB/c mice for a boost with a recombinant modified vaccinia virus Ankara expressing Gag (MVA-Gag). After the boost high frequencies of predominantly Gag-specific CD8^+^ T cells were detected when BCGpan-Gag was the prime in contrast to induction of predominantly Gag-specific CD4^+^ T cells when priming with BCG-Gag. The differing Gag-specific T-cell phenotype elicited by the prime-boost regimens may be related to the reduced inflammation observed with the pantothenate auxotroph strain compared to the parent strain. These features make BCGpan-Gag a more desirable HIV vaccine candidate than BCG-Gag. Although no Gag-specific cells could be detected after vaccination of BALB/c mice with either recombinant BCG vaccine alone, BCGpan-Gag protected mice against a surrogate vaccinia virus challenge.

## Introduction

Information in the 2010 UNAIDS Report on the global AIDS epidemic indicates a global decline in HIV infection, observed as a lower number of infections and deaths from AIDS. However there is a strong warning in the Report that concerted efforts to improve prevention of disease is of paramount importance as this will contribute to prevention of infection. The quest for an HIV vaccine should be an integral component of prevention strategies. The precise requirements of an HIV vaccine needed for eliciting protection against infection are as yet not known. Immune responses to human immunodeficiency virus type 1 (HIV) that participate in virus replication control, as observed in infected individuals termed long-term non progressors or elite controllers, include specific cellular responses that strongly target Gag [1], [2]. Induction of such cellular HIV-specific immune responses is therefore one of the favourable and necessary requirements of a protective vaccination strategy [3]. There is abundant evidence indicating heterologous prime-boost vaccine regimens that combine the use of recombinant bacterial vaccine vectors and recombinant virus vaccine vectors expressing HIV antigens and HIV virus like particle protein vaccines induce robust HIV-specific cellular immune responses [4]–[6].

*Mycobacterium bovis* bacillus Calmette-Guérin (BCG) has been explored as an HIV vaccine vector since the early 1990s [7], [8]. BCG has a record of being safe in that it has been given to billions of people worldwide with a very low incidence of serious complications and importantly it induces long lasting immunity [9]. This knowledge has lead to many endeavours focussing on the use of BCG and related mycobacteria as a live recombinant vaccine vehicle [5]. A variety of viral, bacterial, parasitic and human antigens when expressed in BCG and used in experimental models yielded protective immunity against malaria parasites, Lyme disease, pneumococcal infection, measles, tetanus, cutaneous leishmaniasis, cottontail rabbit papillomavirus, murine rotavirus and listeriosis [10]–[15]. *Mycobacterium smegmatis* has also been explored as a possible HIV vaccine vector and recently a lysine auxotroph of BCG and a BCG strain expressing perfringolysin (AERAS-401), have also been evaluated as possible vectors [16]–[21]. Cell mediated responses as well as antibody responses to the HIV insert, some of which provided protection against challenge, have been detected in murine and macaque models when mycobacterial vectors were used [16], [22], [23]. A major feature of recombinant mycobacterial HIV vaccine vectors is their ability to predominantly prime the immune system for a boost with either recombinant adenovirus, recombinant poxviruses or proteins [5], [6], [16], [17], [20], [21], [24], [25]. The finding that non-human primates primed with rBCG expressing simian immunodeficiency virus (SIV) Gag and boosted with a recombinant replication deficient vaccinia virus expressing the homologous antigen were protected against a challenge with SHIV KS661c has inspired confidence in BCG as a valuable vaccine vector for HIV genes [4].

We have shown that rBCG expressing HIV-1 subtype C Gag is able to prime the immune system of baboons to a boost with Pr55gag virus-like particles (Gag VLPs) with induction of high magnitudes of Gag-specific CD8^+^ and CD4^+^ T cells as well as anti-Gag antibodies [6]. A feature of this vaccine was utilization of an episomal vector with the *M. tuberculosis mtrA* promoter (which is down-regulated in culture and up regulated after uptake by mammalian cells) to express Gag and the 19 kDa leader sequence to direct the Gag protein to the bacterial cell membrane. This vaccine was found to have a degree of genetic instability characterised by deletions and rearrangements of the plasmid and was thus unsuitable for manufacture.

In this study, a new plasmid expressing Gag was constructed. Genetically stable rBCG vaccines expressing Gag, BCG-Gag and BCGpan-Gag were generated using the Pasteur strain of BCG and a pantothenate auxotroph of Pasteur (BCGΔ*panCD*). Pantothenate is a key precursor of coenzyme A and the acyl carrier protein and is essential for many intracellular processes including fatty acid metabolism, cell signalling and synthesis of polyketides and non-ribosomal peptides [26]. A *M. tuberculosis* pantothenate, auxotroph as well a BCG pantothenate auxotroph, have been studied in mice and guinea pigs. Both showed a lack of virulence but protected from challenge with *M. tuberculosis* [27], [28]. BCG can cause disseminated disease in immunocompromised individuals. BCGΔ*panCD* is safer in SCID mice and undergoes limited replication *in vivo* despite persistence within the host. This study evaluates the vaccines BCG-Gag and BCGpan-Gag for induction of Gag-specific T cell responses with and without a MVA-Gag boost. BCGpan-Gag was also tested for its ability to protect mice against a surrogate vaccinia virus expressing Gag (VV-Gag) challenge.

## Results

### Development of a genetically stable rBCG vaccine

**Figure 1:**
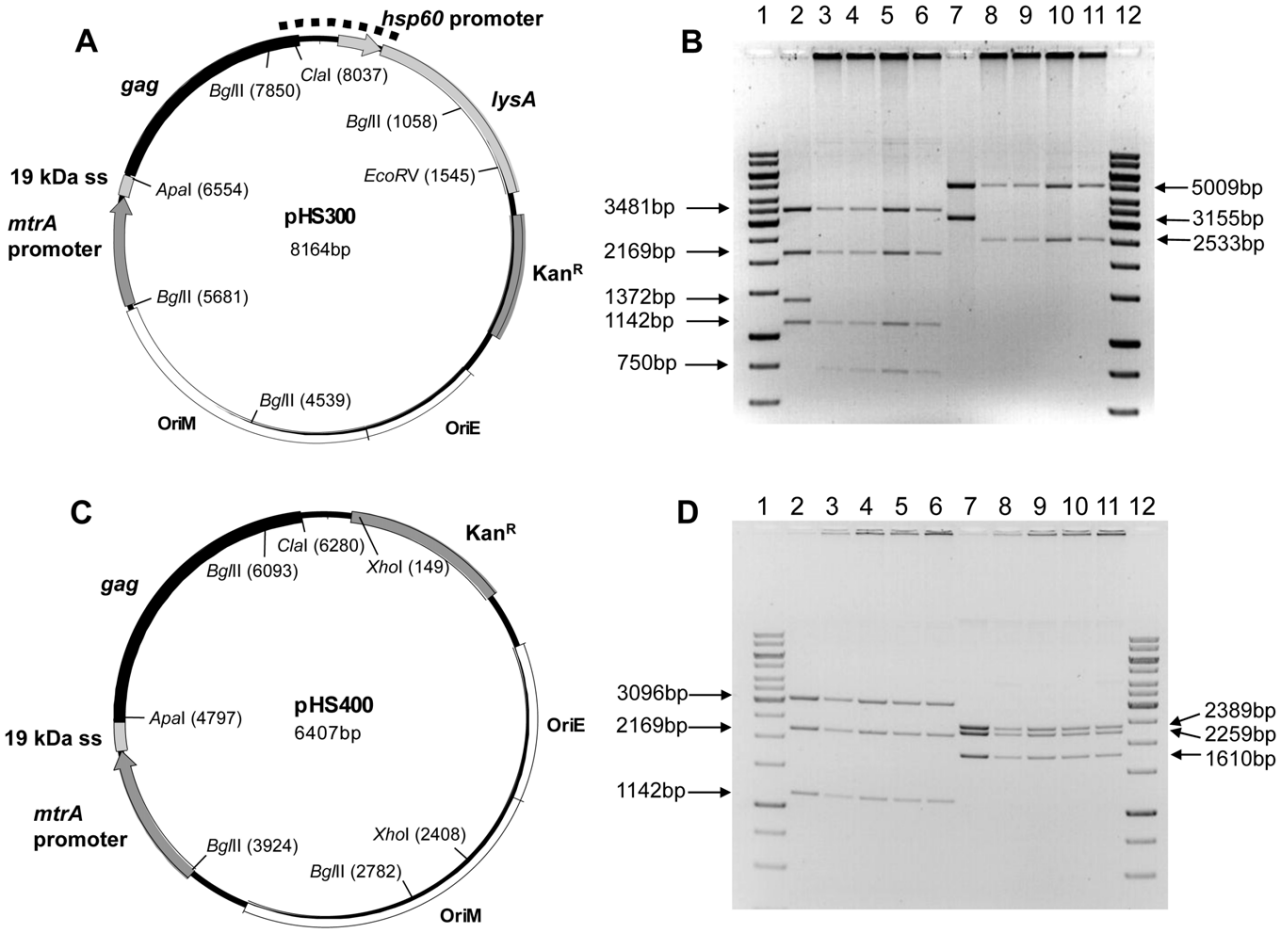
Schematic maps and restriction enzyme digests of pHS300 and pHS400 plasmid DNA isolated from rBCG. (A) Schematic map of plasmid pHS300. The deleted region, found in plasmid DNA isolated from vaccine stocks of rBCG, is indicated by a thick, dotted line. (B) Lanes 2 & 7 contain plasmid DNA prepared prior to transformation into BCG (positive controls). Lanes 3–6 and 8–11 contain pHS300 plasmid DNA isolated from 4 different rBCG[pHS300] transformants. Plasmid DNA in lanes 2–6 was digested with restriction enzyme *Bgl*II and plasmid DNA in lanes 7–11 was digested with restriction enzymes *EcoR*V & *Apa*I. (C) Schematic map of plasmid pHS400. (D) Lanes 2 & 7 contain pHS400 plasmid DNA prepared prior to transformation into BCG (positive controls). Lanes 3–6 and 8–11 contain plasmid DNA isolated from 4 different rBCG[pHS400] (rBCG-Gag) transformants. Plasmid DNA in lanes 2–6 was digested with restriction enzyme *Bgl*II and plasmid DNA in lanes 7–11 was digested with restriction enzymes *Xho*I & *Apa*I. Results were the same for rBCG and rBCGΔ*panCD*.

The effect of codon optimizing the *gag* gene for BCG usage on genetic stability of rBCG was then investigated. BCG was transformed with the plasmids pHS300 and pRT106 expressing codon optimised and uncodon optimised *gag* genes respectively. Colonies produced by BCG transformed with plasmid pHS300 were larger than those transformed with pRT106, indicating that codon-optimisation of the *gag* gene for expression in mycobacteria had probably reduced the metabolic load generated by expression of the viral antigen and thus improved the growth rate of the recombinant BCG. Plasmid DNA isolated from BCG[pHS300] and BCGΔ*panCD*[pHS300] vaccine stocks contained deletions and rearrangements. Restriction mapping and sequencing of plasmid DNA isolated from these vaccines indicated that the p7p1p6 region of the *gag* gene, the *hsp60* promoter and a portion of the *lysA* gene were deleted in all the rBCG analysed (Figure 1 A & B). This region of deletion varied between different vaccine stocks but, in all cases, the *hsp60* promoter was deleted. High levels of LysA protein were detected by SDS-PAGE analysis of the BCG lysates (data not shown). To determine whether the high levels of LysA were contributing to the instability of the rBCG, the *hsp60* promoter and *lysA* gene were deleted from the shuttle vector to generate the plasmid pHS400 (expressing a codon optimised *gag* gene), following which BCG[pHS400] (BCG-Gag) and BCGΔ*panCD*[pHS400] (BCGpan-Gag) were generated. Vaccine stocks were found to be genetically stable and this stability was maintained after *in vitro* passage for 30 generations (Figure 1 C & D). The *in vivo* stability of BCG-Gag and BCGpan-Gag was also confirmed by restriction enzyme mapping of plasmid DNA isolated from rBCG recovered from mice splenocytes 6 weeks post vaccination (data not shown). Growth rates of BCG-Gag and BCGpan-Gag in broth supplemented with pantothenate were comparable to those of the respective non-recombinant BCG strains (data not shown). No growth of BCGΔ*panCD* was observed in the absence of pantothenate confirming its auxotrophic phenotype.

### Cellular responses, bacterial burden and histopathology induced by rBCG vaccines

**Figure 2:**
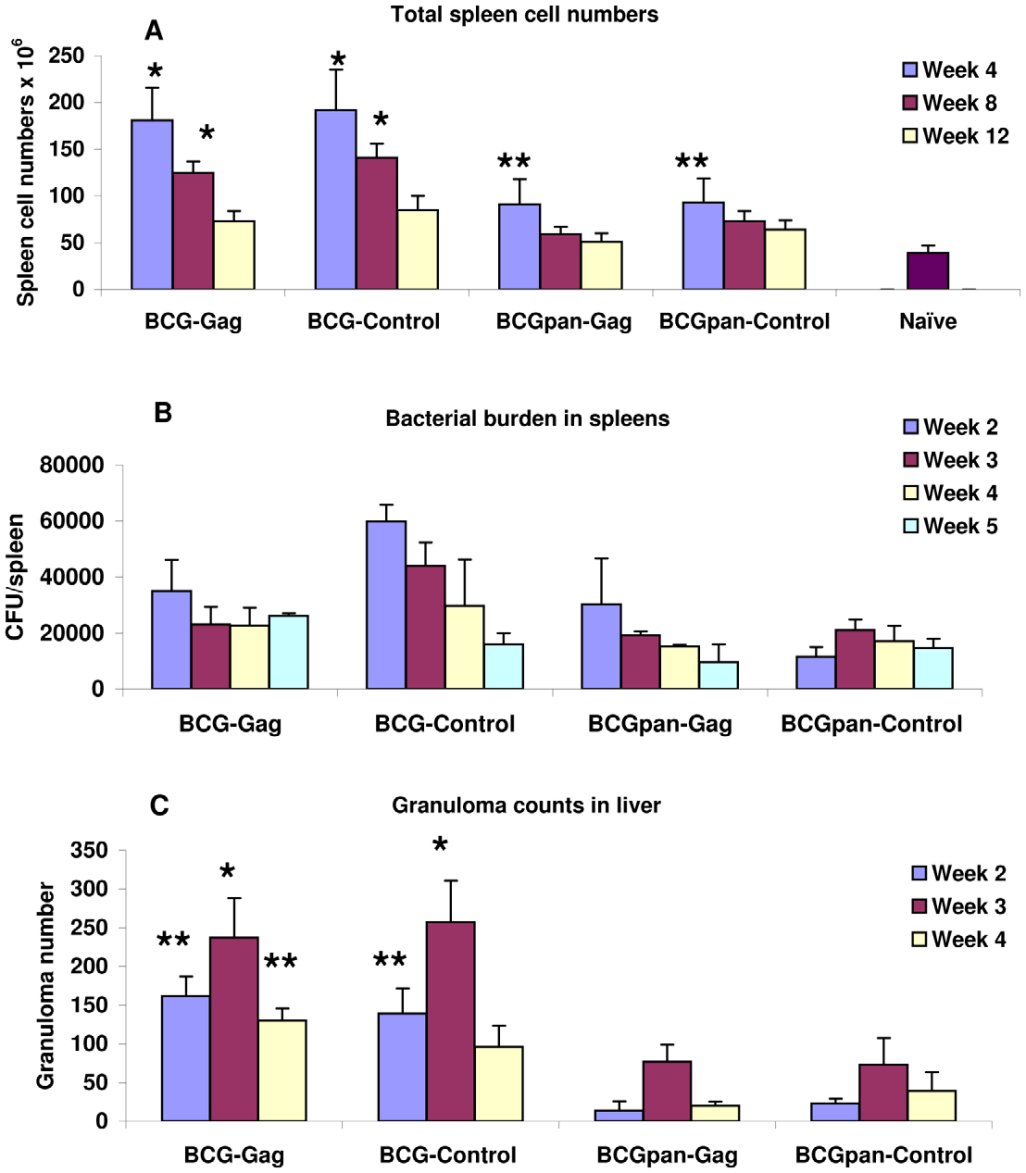
Cellular responses to BCG-Gag and BCGpan-Gag. Groups of mice were vaccinated with BCG-Gag, BCG-Control, BCGpan-Gag or BCGpan-Control (10^7^ cfu, i.p.). (A) Total cell numbers in spleens harvested at the indicated times. Bars are mean total spleen cell numbers and standard deviation of the mean for 10 mice per vaccine. Value for naive mice (unvaccinated) is also indicated. (B) Bacterial burden at the indicated times determined by growth (3 weeks, 37°C) of spleen homogenates on 7H10 medium with kanamycin and also with pantothenate for the BCG*ΔpanCD* strain. Bars are mean CFU per spleen and standard deviation of the mean for 3 mice per vaccine. (C) Quantitative analysis of liver granuloma formation 3 weeks after infection. Bars are the mean and standard deviation of the mean of microscopic counts for 3 mice per vaccine. Counts are from 10 fields per liver section with magnification 100×. This corresponds to 1 mm^2^ per field, total 10 mm^2^, which is approximately the total area of a liver. Asterisks indicate (A) statistical significance compared to naive mice, or (C) compared to *ΔpanCD* strain (C); *<0.01; **<0.05; Student's t-test for unpaired data.

**Figure 3:**
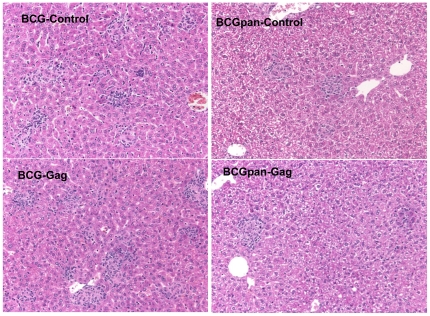
Liver sections of mice vaccinated with rBCG. Histopathology examination of formalin fixed liver sections stained with haematoxylin and eosin showing the frequency of granulomas induced by the vaccines. Magnification 200×, and a representative view from one of 3 mice per vaccine.

Vaccination of mice with the rBCG vaccines (10^7^ cfu, i.p.) resulted in spleen enlargement and an associated increase in the total splenocyte yield compared to that of naive spleens (Figure 2A). At 4 weeks post vaccination with BCG-Gag or a BCG-Control (rBCG not expressing Gag), caused a 4.6–fold increase in splenocyte numbers compared to that of naïve mice (p<0.01). At 12 weeks post vaccination total splenocyte numbers were always higher than that from naive spleens but the differences were not statistically significant. The splenocyte yield in response to BCGpan-Gag or BCGpan-Control (rBCGΔ*panCD* not expressing Gag), vaccination was 2-fold greater (p<0.05) than that from naive spleens at 4 weeks but not at 8 and 12 weeks post vaccination (Figure 2A). The proportion of CD3^+^ cells in the spleens following vaccination with BCG-Gag or the control rBCG as determined by flow cytometry was consistent at 41±8% (n = 10) but not significantly different from the proportion of CD3^+^ cells (39±4%, n = 10) in spleens from mice vaccinated with BCGpan-Gag or the control rBCG. These CD3^+^ cell proportions post rBCG vaccination compare with those from naïve spleens which have a CD3^+^ cell proportion of 43±8% (n = 10). Importantly, the CD4^+^:CD8^+^ T cell ratio of the rBCG vaccinated mice was the same as that of naive mice, 2.4±0.3 (n = 10). Bacterial numbers in the spleen after the rBCG vaccination declined over time and there was no significant difference (p>0.05) in bacterial numbers for the vaccines at any of the observed times (Figure 2B). Histopathology analysis of liver sections 3 weeks post vaccination revealed BCG-Gag induced a significantly larger number of granulomas (p<0.05, week2; p<0.01, week 3) that were frequently larger and contained more inflammatory cells than those isolated from mice vaccinated with BCGpan-Gag (Figure 2C & 3). For both BCG-Gag and BCGpan-Gag granulomas were well formed (Figure 3). Overall the inflammation induced by the wild type BCG was characterised by more prominent cellular infiltration and hepatocyte damage than that induced by the *ΔpanCD* strain (Figure 3).

### rBCG vaccines expressing Gag prime the immune system for a boost with MVA-Gag

**Figure 4:**
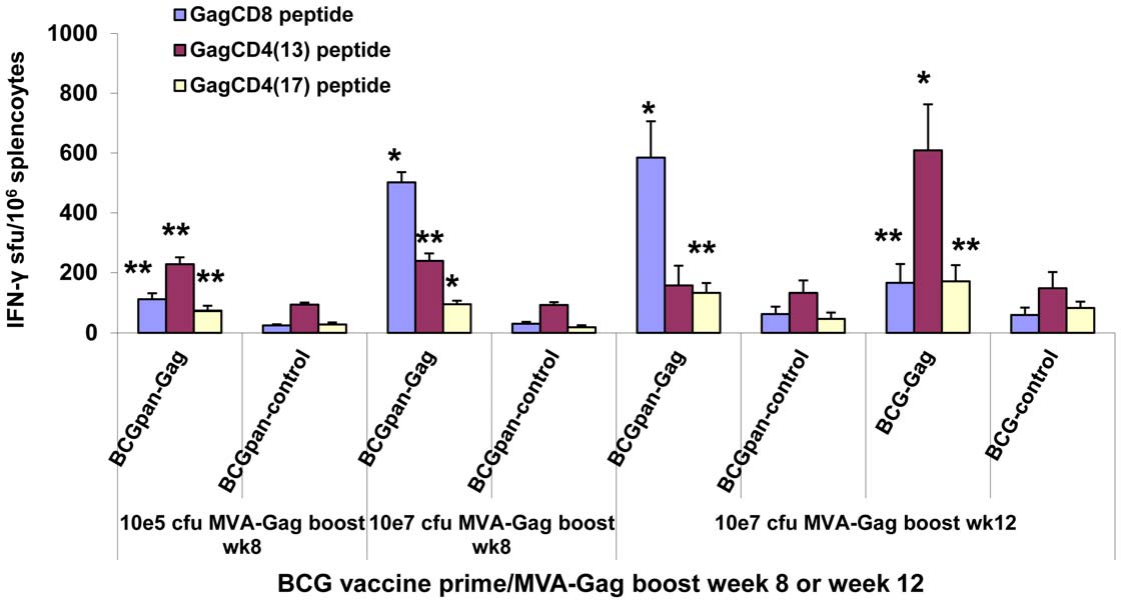
IFN-γ ELISPOT responses induced by a prime with the rBCG vaccine and MVA-Gag boost. Groups of mice were primed with BCG-Gag (10^7^ cfu) and BCG-Control (10^7^ cfu), BCGpan-Gag (10^7^ cfu or 10^5^ cfu) or BCGpan-Control (10^7^ cfu or 10^5^ cfu) then boosted with MVA-Gag (10^7^ pfu, i.m.) at week 8 or week 12. Spleens were harvested 12 days after the boost, pooled from 5 mice per group and used in an IFN-γ ELISPOT assay with the indicated Gag-specific CD8^+^ or CD4^+^ peptides. Bars indicate the mean and standard deviation of the mean IFN-γ ELISPOT response to an individual Gag CD8^+^ or Gag CD4^+^ peptide for 3 separate experiments. Data are responses after subtraction of background responses which were not more than 20 sfu/10^6^ splenocytes. Asterisks indicate statistical significance of the mean IFN-γ ELISPOT responses to the individual Gag peptides obtained for a rBCG vaccine and MVA-Gag boost compared to that for the respective control rBCG vaccine prime MVA-Gag boost; *<0.01; **<0.05; Student's t-test for means of unpaired data.

Mice were vaccinated with the individual BCG vaccines (10^5^ cfu or 10^7^ cfu) and Gag-specific immune responses investigated using the IFN-γ ELISPOT assay to detect individual responses to a Gag CD8^+^ peptide and two Gag CD4^+^ peptides, GagCD4(13) and GagCD4(17). No Gag-specific T cell responses were detected at weekly intervals up to 12 weeks after vaccination. To detect possible priming of the immune system by these rBCG vaccines, groups of mice were primed on day 0 with the BCG vaccines then boosted at week 8 or week 12 with MVA-Gag and Gag peptide-specific immune responses were detected 12 days after the boost (Figure 4).

BCGpan-Gag at a dose of 10^5^ cfu or 10^7^ cfu unlike BCG-Gag, primed for a MVA-Gag boost given at week 8 (Figure 4). Mean cumulative Gag-specific IFN-γ ELISPOT responses (3 separate experiments) of 412±23 sfu/10^6^ splenocytes were observed for a prime with 10^5^ cfu BCGpan-Gag; and 835±34 sfu/10^6^ for a prime with 10^7^ cfu BCGpan-Gag. These mean cumulative Gag peptide responses were 2.8 fold (p<0.05) and 5.8 fold (p<0.01) greater respectively than for a respective control BCG prime and MVA-Gag boost (Figure 4). Both Gag CD4^+^ and CD8^+^ peptide responses were boosted with Gag-specific CD8^+^ cells contributing 36% and 60% to the total mean cumulative response for a dose of 10^5^ cfu or 10^7^ cfu BCGpan-Gag respectively. In comparison the contribution of Gag-specific CD8^+^ cells to the total cumulative response for the control BCG prime (10^5^ cfu or 10^7^ cfu) and MVA-Gag boost was 17% and 22% respectively (Figure 4).

BCG-Gag did prime the immune system to a MVA-Gag boost given at week 12 after the BCG vaccine prime when the priming dose was increased to 10^7^ cfu. Comparison of Gag peptide responses when the MVA-Gag boost was given at week 12 after a prime with either BCGpan-Gag (10^7^ cfu) or BCG-Gag (10^7^ cfu) is shown in Figure 4. For both vaccines Gag CD8^+^ and CD4^+^ peptide responses were boosted and the cumulative mean response (3 independent experiments) to the Gag peptides in the IFN-γ ELISPOT assay for either a prime with BCG-Gag or BCGpan-Gag were similar, 946±212 sfu/10^6^ splenocytes and 847±142 sfu/10^6^ splenocytes respectively. These cumulative Gag-peptide responses were approximately 3.4 fold higher (p<0.01) than the responses for a control BCG vaccine prime/MVA-Gag boost (Figure 4). However, when BCG-Gag was the priming vaccine, Gag-specific CD4^+^ cells contributed 82% and Gag-specific CD8^+^ cells 18% to this cumulative response, indicating MVA-Gag predominantly boosted Gag-specific CD4^+^ cells. In contrast when BCGpan-Gag was the prime Gag-specific CD4^+^ contributed 33% and Gag-specific CD8^+^ cells 67% to the cumulative Gag-specific response indicating MVA-Gag predominantly boosted Gag-specific CD8^+^ cells (Figure 4). The contribution of Gag-specific CD8^+^ cells to the cumulative Gag-specific response to a prime with both the control BCG vaccines followed by a boost with MVA-Gag was approximately 24% (Figure 4).

### The rBCG prime and MVA-Gag boost vaccination regimen induced high levels of Gag-specific IFN-γ, TNF-α and IL-6

**Figure 5:**
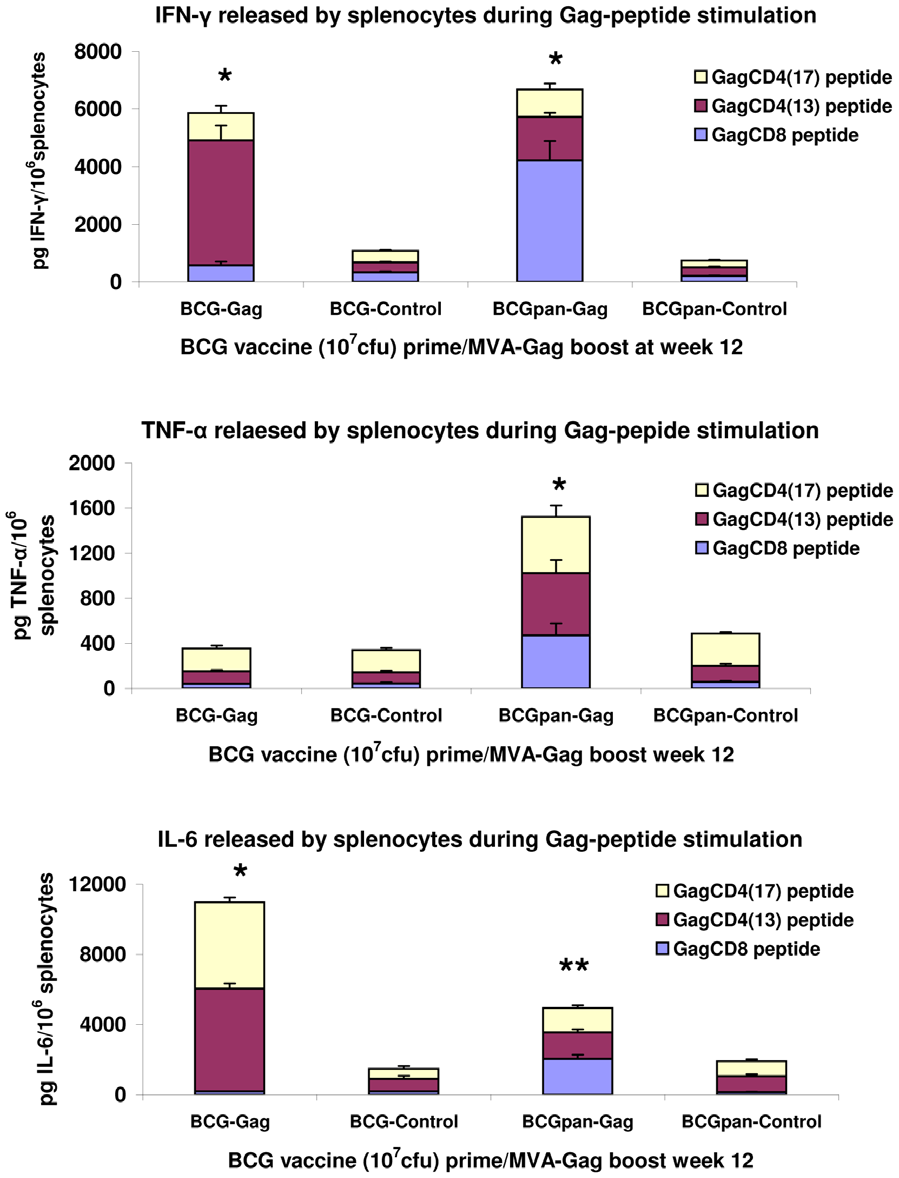
Gag-specific IFN-γ, TNF-α and IL-6 responses induced by a rBCG prime and MVA-Gag boost. Groups of mice were vaccinated with10^7^ cfu BCG-Gag, BCG-Control, BCGpan-Gag or BCGpan-Control then boosted with MVA-Gag (10^7^ pfu) at week 12. Spleens were harvested 12 days after the boost and pooled from 5 mice per group and stimulated in culture with the indicated Gag-specific CD8^+^ and CD4^+^ peptides. Bars indicate the Gag peptide-specific cumulative cytokine response and blocks within each bar indicate the mean and standard deviation of the mean cytokine response for 3 individual experiments to either a Gag CD8^+^ or Gag CD4^+^ peptide. All responses are after subtraction of background responses of not more than 2 pg per 10^6^ splenocytes. Asterisks indicate statistical significance of the cumulative cytokine response for the rBCG vaccine compared to the control rBCG vaccine. *<0.01; **<0.05; Student's t-test for unpaired data.

A cytokine bead array assay quantified the level of IFN-γ, TNF-α and IL-6 produced by splenocytes after a prime with the BCG vaccines and a boost with MVA-Gag given at week 12 after the BCG vaccination (Figure 5). High levels of IFN-γ were released from splenocytes in response to Gag peptide stimulation (Figure 5A). When BCG-Gag was the priming vaccine 10% of the total Gag-specific IFN-γ production of 5895±473 pg/10^6^ splenocytes (3 individual experiments) was from CD8^+^ cells, while for prime with BCGpan-Gag, CD8^+^ cells contributed 63% to the total Gag-specific IFN-γ production of 6673±328 pg/10^6^ splenocytes (3 individual experiments). These levels of IFN-γ produced by Gag peptide stimulated splenocytes were more than 6 fold greater (p<0.01) than those for a prime with the control BCG vaccines followed by a MVA-Gag boost (Figure 5A). No Gag-specific TNF-α above background responses could be detected for a BCG-Gag prime and MVA-Gag boost. This is in contrast to splenocytes from BCGpan-Gag primed and MVA-Gag boosted mice that produced a total of 1532±218 pg TNF-α/10^6^ splenocytes (3 individual experiments) when stimulated with Gag-peptides, with 31% being produced by Gag-specific CD8^+^ cells. This TNF-α production was four times that of a prime with the BCGpan-Control vaccine (Figure 5B). Splenocytes from BCG-Gag primed and MVA-Gag boosted mice produced 10771±520 pg IL-6 and all from Gag-specific CD4^+^ cells (3 individual experiments). This Gag-specific IL-6 production was 8-fold above (p<0.01) that of a prime with the BCG-Control vaccine. A prime with BCGpan-Gag elicited 4945 pg IL-6/10^6^ splenocytes with approximately equal quantities from Gag-specific CD8^+^ and CD4^+^ cells. This Gag-specific IL-6 production was 3-fold above (p<0.05) that of a prime with the BCGpan-Control vaccine (Figure 5C).

### BCGpan-Gag protects against a VV-Gag challenge

**Figure 6:**
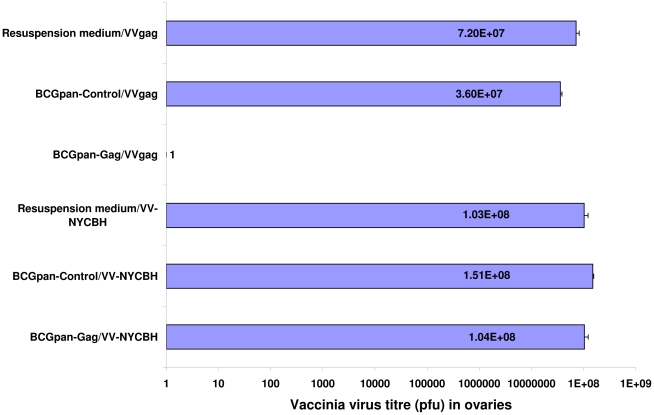
Protection from a Vaccinia Virus-Gag challenge by BCGpan-Gag. Challenge of mice vaccinated with BCGpan-Gag or BCGpan-Control vaccine with VV-Gag or VV-NYCBH. Groups of 10 mice were vaccinated with BCGpan-Gag (2×10^6^ cfu), BCGpan-Control (2×10^6^ cfu) or resupension medium on day 0 and day 28. Two weeks later half the mice in each group were challenged with VV-Gag or VV-NYCBH (1×10^6^ pfu) and ovaries harvested 5 days later to determine virus titres. Bars indicate the mean and standard deviation of the mean virus titre.

The data from the immune assays suggest BCGpan-Gag primes predominantly Gag- specific CD8^+^ cells. Thus the ability of BCGpan-Gag to protect mice against a VV-Gag challenge was assessed. Similar titres of VV-NYCBH were measured in mice that were challenged with this control vaccinia virus after vaccination with either the BCGpan-Gag, the BCGpan-Control vaccine or the resuspension medium. This indicates that neither the rBCG vaccines nor the resuspension medium provided nonspecific vaccinia protection. In addition no non-specific protection against VV-Gag was observed in mice vaccinated with the BCGpan-Control vaccine or the BCG vaccine resuspension medium as similar VV-Gag titres were obtained after a VV-Gag challenge (Figure 6). However for mice vaccinated with BCGpan-Gag then challenged with VV-Gag, no virus was recovered from the ovaries, which translates to >7 log protection compared to that of a vaccination with the BCGpan-Control or the BCG vaccine resuspension medium followed by VV-Gag challenge.

## Discussion

In this study BCG has been evaluated as an HIV vaccine vector expressing Gag. The *ΔpanCD* BCG auxotroph was compared to the wild type BCG as a vaccine vector as this strain has been shown to be less virulent than the wild type strain in mice and guinea pigs. The observation that BCG vaccination of HIV positive children has accounted for mycobacterial disease in a proportion of these immunocompromised subjects strongly indicates the need for the development of safer mycobacterial vectored vaccines [29], [30]. Second generation auxotroph strains of *M. tuberculosis* and BCG have thus been generated for use against tuberculosis. Deletion of genes important for mycobacterial growth such as *lysA*, *leuD*, *met* and *panCD* results in attenuated growth *in vivo* with a consequent improved safety profile in immunocompromised animals and concomitant enhanced or comparable protection of mice and guinea pigs against *M. tuberculosis* challenge compared to the parent strain [28], [31]–[34]. rBCG expressing listeriolysin or perfringolysin O have also been shown to have improved safety in SCID mice as compared to the wild type strain [13], [35]. Safety issues are also of concern with the use of BCG as a HIV vaccine vector and this has led to auxotroph BCG strains being considered as alternative vectors for immuocompromised individuals [28]. These mutant strains do not show compromised immunogenicity in animal studies [20], [24], [36]. The *ΔpanCD* BCG auxotroph has previously been shown to require pantothenate supplementation to grow in human macrophages and is nonpathogenic in SCID mice. When used to express the *M. tuberculosis* 30 kDa major secretory protein in the development of a candidate TB vaccine, rBCG(*panCD*)30, this auxotroph was better tolerated at higher doses than the parent strain and provided protection comparable to that of BCG in guinea pigs [28]. *In vitro* our BCGpan-Gag vaccine grew at a rate similar to BCG-Gag in the presence of pantothenate supplementation in broth culture. Deletion of the *hsp60* promoter and *lysA* gene from the vector resulted in genetically stable BCG and *ΔpanCD* BCG auxotroph vaccines, both *in vitro* and *in vivo*. No plasmid deletions and rearrangements were observed. This modification of the plasmid together with the use of a codon optimised *gag* gene was essential to prevent deletion of p7p1p6 from the C-terminus of Gag. Plasmid instability has previously been shown to be associated with the activity of the strong *hsp60* promoter [37]–[39]. It is important to have a vaccine that includes p7p1p6 as HIV infected individuals do mount immune responses to this Gag region [40]. The BCG codon optimised *gag* gene was used to enhance translational activity. The observed genetic stability of our vaccines may be attributed to two factors, fusion of the *gag* gene to the *M. tuberculosis* 19 kDa signal sequence, to assist with transport of the Gag protein to the surface of the mycobacterium, and control of HIV-1 *gag* gene expression by the *M. tuberculosis mtrA* promoter, which is up-regulated after uptake of rBCG by phagocytosing cells [6], [37], [41]–[43]. *In vivo* expression of HIV-1 Gag was not assessed in this study, however both BCG-Gag and BCGpan-Gag primed the immune system for a MVA-Gag boost, indicating that Gag presentation must have occurred after cellular uptake of the rBCG vaccines.

Comparison of immune responses to Gag induced by BCG-Gag and BCGpan-Gag indicated that although no Gag-specific T cells could be detected in response to the vaccines alone, Gag-specific responses were detected after the MVA-Gag boost. These responses were not due to BCG-dependent adjuvant activity as Gag-specific T cell responses induced by a MVA-Gag vaccination alone were not significantly different from that observed after a prime with the BCG-Control or BCGpan-Control vaccines and a MVA-Gag boost (data not shown). Thus both vaccines did appear to prime the immune system.

In addition BCGpan-Gag induced Gag-specific responses that protected against a surrogate vaccinia virus-Gag challenge. Greater than 7 logs of protection were seen in the vaccinia virus challenge when mice were immunised with BCGpan-Gag. Protection from challenge in this model is associated with CD8^+^ responses [44] and therefore this result indicated that the HIV-1 specific CD8^+^ T cell immune response induced with BCGpan-Gag was protective.

Data from several studies indicate mycobacterial vaccines prime the immune system for a booster vaccine [5], [6], [17], [19], [20], [24]. Although a pro-apoptotic *M. tuberculosis ΔlysAΔsecA2* mutant and a BCG Pasteur *lysA* auxotroph have generated CD8^+^ cells to the HIV plasmid insert, an inability to detect responses or detection of low responses to an HIV or SIV insert delivered by BCG or BCG and *M. tuberculosis* single and double auxotrophs have generally been reported in murine and non-human primate vaccine studies [19], [36]. Mechanisms of immune system priming by BCG are associated with *in vivo* replication rates and levels and duration of antigen expression in the bacteria [45]. BCG replication after vaccination is slow which favours low levels of antigen expression followed by low levels of antigen presentation [46]. Subsequently low magnitudes of induced antigen specific T cells that differentiate to the memory phenotype soon after vaccination are generated and are stimulated by the booster vaccine [47].

The responses to a MVA-Gag boost that we detect after a prime with BCG-Gag or BCGpan-Gag indicate Gag-specific T cells were probably induced by the BCG vaccines and it is possible the memory phenotype of these cells prevented their detection in the IFN-γ ELISPOT assay [47]. Gag-specific CD4^+^ cells were predominantly generated to the boost after a BCG-Gag prime. In contrast BCGpan-Gag primed for a boost of predominantly Gag-specific CD8^+^ cells. These vaccines were given at a dose of 10^7^ cfu per mouse. MVA-Gag boosted primary Gag-specific CD8^+^ and CD4^+^ cells as early as 8 weeks post the BCGpan-Gag prime; but this was not achieved with a BCG-Gag prime. The overall greater inflammation generated by the wild type strain may have prevented the development of the secondary responses. These Gag-specific T cells produced IFN-γ, TNF-α as well as IL-6, cytokines which are proposed to be necessary for control of HIV infection [48]–[50]. Induction of a Gag-specific CD4^+^ response may be expected as BCG per se induces CD4^+^ cell responses in mice which would influence predominant insert-specific CD4^+^ cell development [51]. However a CD4^+^ response does assist CD8^+^ development [52], [53]. Induction of Gag-specific CD4^+^ and CD8^+^ cells by the BCGpan-Gag prime and MVA-Gag boost regimen is a required favourable vaccine response and suggests both MHC class I and II presentation of the antigen occurs with generation of CD8^+^ cells a consequence of possible efficient cross priming by the BCG vaccine.

As inflammatory signals from BCG are expected to also influence the characteristics of the immune response to the HIV insert the reduced inflammation caused by the BCG*ΔpanCD* strain may account for the immune response to the *gag* gene seen as both Gag-specific CD8^+^ and CD4^+^ cells [48]. This modulation of inflammation by the BCG*ΔpanCD* strain vaccination was observed in this study as a reduction in early expansion of CD3^+^ cells as well as a reduction in granuloma size and number compared to the parent strain. Despite this lower cellular response to the BCG*ΔpanCD* strain, bacterial replication was similar to that of the wild-type strain. Similar reduced pathology with constraint of bacterial growth has been observed in mice in response to a *M. tuberculosis ΔpanCD* mutant as well as the *M. tuberculosis whiB3* and *sigH* mutants [33], [54], [55]. Deletion of the *panC* and *panD* genes inhibits the synthesis of pantothenate which is one of the requirements for protection of bacteria from oxidative stress. In addition there is a decrease in phospholipid and intermediate amino acid and polyketide biosynthesis through deletion of the *panC* and *panD* genes. This lack of pantothenate biosynthesis associated with decreased inflammation may play a major role in the orchestration of immune responses to Gag expressed by the BCGpan-Gag vaccine.

In conclusion this is the first study using the BCG *ΔpanCD* auxotroph as an HIV vaccine vector and shows that, when used as a vector for the *gag* gene, HIV-specific T cells are induced that protect against vaccinia virus surrogate challenge and can be boosted to a high level with a heterologous booster vaccine. The reduced inflammation induced by the auxotroph appears to be an important factor in the generation of this broad immune response to the HIV insert. Thus, the BCGpan-Gag vaccine appears, through its combined features of genetic stability, safety in immunocompromised mice and ability to elicit CD8^+^ and CD4^+^ T cell responses in heterologous prime boost vaccine regimes, to be a more promising HIV vaccine candidate than BCG-Gag.

## Methods

### Construction of recombinant BCG (rBCG) expressing Gag and vaccine preparation

Wild type *M. bovis* BCG Pasteur1172 P2 (BCG) (supplied by the Statens Seruminstitut, Denmark) and *M. bovis* BCG mc^2^6000 (BCGΔ*panCD*), a pantothenic acid auxotroph strain derived from BCG Pasteur (constructed as per the *M. tuberculosis* Δ*panCD* mutant [33]), were grown on Middlebrook 7H10 agar supplemented with 10% oleic acid-albumin-dextrose-catalase (OADC) and 0.5% glycerol (7H10) or in Middlebrook 7H9 broth supplemented with 10% OADC, 0.2% glycerol and 0.025% tyloxapol (7H9) on rollers (4 rpm) at 37°C. Kanamycin (10 µg/ml) was included in the media for plasmid selection where required. Media was supplemented with pantothenate (48 µg/ml) and hygromycin (50 µg/ml) for the growth of BCGΔ*panCD*.

The shuttle vector pHS300 expressing HIV-1 Gag was constructed as follows: the full length HIV-1 subtype C *gag* gene [56] was codon optimised for use in BCG and cloned into the *Apa*I and *Cla*I restriction sites of plasmid pCB119, thus fusing the *gag* gene to the nucleotides encoding the *M. tuberculosis* 19 kDa signal sequence and placing it under the control of the *M. tuberculosis mtrA* promoter (Figure 1). Plasmid pHS400 was derived by deletion of the *hsp60* promoter and *lysA* gene from vector pHS300 (Figure 1).

The shuttle vectors pHS300, pHS400, pRT106 [6] and pCONEPI (vector not containing *gag*, Genbank accession DQ191755) were introduced into BCG and BCGΔ*panCD* by standard mycobacterial electroporation procedures to generate rBCG [57]. The rBCG were plated on 7H10 media plus kanamycin (10 µg/ml) with the appropriate supplements and incubated at 37°C for approximately 3 weeks. Vaccine stocks were prepared by culturing selected recombinants in 5 ml 7H9 media, then inoculating 100 ml 7H9 media in a roller bottle and growing cells until mid-logarithmic phase (∼OD_600_ 0.8). The cultures were then harvested and the pellets resuspended in resuspension medium (0.85% NaCl; 10% glycerol; 0.025% tyloxapol) to give a final OD_600_ = 10 which is equivalent to a concentration of 1×10^9^ cfu/ml. The vaccine stocks were stored at −80°C till required. To confirm *in vitro* genetic stability plasmid DNA was recovered from vaccine stocks and mapped with restriction enzymes and the HIV-1 *gag* gene was sequenced. Prior to vaccination the vaccines were defrosted on ice and passed through a 21 gauge syringe needle 10 times in order to disperse clumps just prior to injection.

### *In vitro* and *in vivo* stability of the rBCG

To assess stability of the rBCG, cultures were passaged daily for approximately 30 generations in liquid media with and without antibiotic selection. Aliquots of the culture were frozen at −80°C in 15% glycerol prior to each passage. The number of colony forming units obtained after plating suitable dilutions of the cultures on 7H10 media with and without kanamycin (10 µg/ml) were compared to determine plasmid stability. To assess the genetic stability of the rBCG, plasmid DNA was recovered after 30 generations and mapped with restriction enzymes (Figure 1). To determine *in vivo* plasmid stability, plasmid DNA was isolated from mycobacterial colonies and mapped with restriction enzymes 6 weeks post vaccination.

### Recombinant MVA expressing Gag (MVA-Gag)

MVA-Gag expressing a matching Gag antigen was used as a booster. HIV-1 *gag* was inserted into the Del III region of the MVA virus genome under the transcriptional control of the modified-H5 promoter [58]. MVA-Gag was grown on the chorioallantoic membranes (CAMs) of 10–12 day old chick embryos and harvested after 72 hours. Titration was performed in BHK-21 cells, using rabbit anti-vaccinia antibody (Biogenesis, Poole, UK) and swine anti-rabbit HRP (Dako, Glostrup, Denmark) to detect MVA; and sheep anti-p24 antibody (Aalto, Dublin, Ireland) and anti-sheep HRP (Dako, Glostrup, Denmark) to detect Gag. Plaques were visualised by the reaction of peroxidase with o-dianisidine (Sigma, St Louis, USA) in the presence of H_2_O_2_ and counted to determine the virus titre. Irrespective of the antibody used to detect the plaques identical virus titres were obtained indicating MVA-Gag to be stable.

### Vaccinia viruses (VV) and cells

Vaccinia virus expressing a matched HIV-1 Du422 Gag subtype C antigen, vT369, (VV-Gag), was manufactured by Quality Biological (Gaithersburg, MD, USA) and Therion Biologics, Inc (Cambridge, USA). The New York City Board of Health (NYCBH) strain of vaccinia, Vaccinia NYCBH, was used as a control vaccinia (VV-NYCBH). Both viruses were obtained through the AIDS Research and Reference Reagent Program, Division of AIDS, NIAID, NIH. These viruses were amplified on chick chorioallantoic membranes (CAMs) and titrated in CV-1 cells (Highveld Biological, Johannesburg, SA).

### rBCG mouse vaccinations and MVA-Gag boost

The vaccination schedule and all the procedures using female BALB/c mice (8–10 weeks old in groups of 5) were approved by the UCT Animal Ethics Committee (reference UCTAEC 01-041) and performed by a trained animal technologist. The BCG vaccine and doses used were either 10^5^ cfu or 10^7^ cfu given via the intraperitoneal (i.p.) route in 200 µl resuspension medium. For mice that were primed with the rBCG vaccines and then boosted with MVA-Gag, the boost was given at week 8 or 12 after the prime, as an intramuscular (i.m.) vaccination of 10^7^ pfu MVA-Gag in 100 µl PBS with 50 µl injected into each quadriceps muscle.

### Bacterial burden and histopathology

Bacterial growth was determined weekly from week 2 to week 5 post vaccination (10^7^ cfu, i.p.). Spleens from individual mice were collected in resuspension medium (2 ml) and homogenized. Ten-fold serial dilutions of the homogenate were plated on 7H10 agar containing the appropriate supplements in the presence or absence of kanamycin (10 ug/ml). Colonies were counted after incubation at 37°C for approximately 3 weeks.

For histopathology studies livers from individual mice were harvested at weekly intervals after vaccination (10^7^ cfu, i.p.) and fixed overnight in 10% phosphate buffered formalin, embedded in paraffin, sectioned and stained with haematoxylin and eosin. Micrographs were done with an Olympus microscope.

### Preparation of splenocytes for immune assays

Spleens were harvested and pooled from 5 mice per group at weekly intervals after vaccination with the individual BCG vaccines or 12 days after the MVA-Gag boost to determine Gag-specific immune responses. A single cell suspension of splenocytes was prepared then treated with erythrocyte lysing buffer (0.15 M NH_4_Cl, 10 mM KHCO_3_, 0.1 mM Na_2_EDTA) for 1 min at room temperature before suspension in R10 culture medium (RPMI with 10% heat inactivated fetal calf serum (FCS) containing 15 mM β-mercaptoethanol, 100 U penicillin and 100 µg streptomycin/ml, (Invitrogen, Carlsbad, California, USA)). Splenocyte aliquots were stained with anti-CD3^+^ APC, anti-CD4^+^ FITC and anti-CD8^+^ per CP labeled antibodies (BD Biosciences, The Scientific Group, Johannesburg, SA) to determine the proportion of CD3^+^ cells and CD4^+^ and CD8^+^ subpopulations by flow cytometry.

### IFN-γ ELISPOT assay

The Mouse IFN-γ ELISPOT set (BD Pharmingen, The Scientific Group, Johannesburg, SA) was used as per manufacturer's instructions. Splenocytes were plated at 1×10^5^/well in a 200 µl final volume of R10 alone (to determine background response) or medium containing an individual peptide (>95% HPLC pure, Bachem AG, Bubendorf Switzerland) with amino acid sequence matching BALB/c CD8^+^ and CD4^+^ epitopes in Gag, at a concentration of 4 ug/ml. The amino acid sequences of the peptides were AMQMLKDTI (GagCD8^+^ peptide) and NPPIPVGRIYKRWIILGLNK (GagCD4(13) peptide) and FRDYVDRFFKTLRAEQATQE (GagCD4(17) peptide) [59]–[61]. The reaction was stopped after 22 hours incubation at 37°C in 5% CO_2_, and spots were reacted with the detection antibody then developed with Nova Red as per the kit instructions. Spots were counted and analysed using an automatic ELISPOT reader (CTL technologies, Cleveland, Ohio) and Immunospot Version 3.2 software. Average spot numbers were calculated for triplicate reactions. For all experiments the coefficient of variation of the average (standard deviation (SD) of the average expressed as a percentage of the average spot numbers) was not more than 9%. Average spot numbers for responses to peptides that were twice that of average background spot numbers (absence of peptide) were considered positive. Values below this cut off were set to zero. Positive spot numbers were then adjusted to spot forming units (sfu) per 10^6^ splenocytes after background subtraction (not more than 20 sfu/10^6^ splenocytes). The sfu per 10^6^ splenocytes for an individual Gag peptide that arose from either a BCG-Gag or BCGpan-Gag prime and a MVA-Gag boost, was considered a positive peptide response arising from the prime-boost regimen if it was ≥1.5 fold the individual Gag peptide response for a prime with the control BCG (BCG-Control or BCGpan-Control) and a MVA-Gag boost. The sum of sfu per 10^6^ splenocytes for responses to the individual Gag peptides is referred to as the cumulative Gag peptide response.

### Quantification of secreted cytokines

Splenocytes at a concentration of 7.5×10^6^ per ml R10 culture medium were cultured (48 h at 37°C in 5% CO_2_ ) in the absence of peptide (to detect background cytokine release) or with the individual peptides as used in the IFN-γ ELISPOT assay at 4 µg/ml. The cytokine content of culture supernatants were assayed using a cytokine bead array assay (BD Pharmingen, The Scientific Group, Johannesburg, SA) that detected IFN-γ, TNF-α, IL-6 and IL-10. The average of triplicate values was calculated and expressed as pg cytokine per 10^6^ splenocytes. The coefficient of variation of the average value (SD of the average expressed as a percentage of the average) was not more than 7%. Cytokine values were considered positive if greater than twice background values (not more than 2 pg per 10^6^ splenocytes for all assays) and are reported after background subtraction. Values below the cut off were set to zero. No IL-10 above background levels could be detected in the culture supernatant from peptide-stimulated splenocytes for any of the vaccine regimens. The sum of cytokine values obtained with the individual Gag peptide stimuli (cumulative cytokine response) for a prime with either BCG-Gag or BCGpan-Gag and a MVA-Gag boost were considered to be a positive prime-boost response if ≥1.5 fold the cumulative response for a prime with the control BCG (BCG-Control or BCGpan-Control) and a MVA-Gag boost.

### Vaccinia virus challenge

Groups of 10 mice were vaccinated with BCGpan-Gag, BCGpan-Control (2×10^6^ cfu, i.p.) or the resuspension medium used for the rBCG vaccines on day 0 and day 28. Two weeks later half the mice in each group were challenged with either VV-Gag or VV-NYCBH (1×10^6^ pfu, i.p.). Five days after the challenge, ovaries were collected and pooled from 5 mice per group into McIlvains buffer. Pooled ovaries were finely chopped and ground in tenbrook grinders, and cell debris pelleted by low speed centrifugation. The virus in the supernatant was titrated in CV-1 cells, using serial 10-fold dilutions. Thirty six hours post infection the cells were stained with Carbol Fuschin and plaques were counted.

### Statistical analysis

Results are expressed as mean and standard deviation of the mean. Data was statistically analysed using Student's *t* test and *p* values of <0.05 were considered significant.
